# Development of a self-replicating plasmid for non-toxic expression of CRISPR-repressors to study schizophrenia-risk genes

**DOI:** 10.1192/j.eurpsy.2022.408

**Published:** 2022-09-01

**Authors:** D. Abashkin, A. Kurishev, D. Karpov, V. Golimbet

**Affiliations:** Mental Health Research Center, Clinical Genetics Laboratory, Moscow, Russian Federation

**Keywords:** causative genes, schizophrénia, CRISPR editors

## Abstract

**Introduction:**

Genome-wide association studies revealed that polymorphisms located within non-coding regions significantly contribute to the genetic architecture of schizophrenia. Such regions may affect the expression of tens and hundreds of neuronal genes. Epigenetic CRISPR editors help to elucidate the causative polymorphisms. However, efficient CRISPR-repressors are highly toxic to neuronal cells, and their activity rapidly declines with time after transfection due to plasmid silencing. Therefore, less toxic, effective, and long-acting epigenetic CRISPR instruments are required to advance schizophrenia genetic research.

**Objectives:**

We aimed at creating a less toxic and effective CRISPR-repressor for the investigation of schizophrenia-risk genes.

**Methods:**

Plasmids were obtained used standard molecular cloning techniques and lipofected into the SH-SY5Y cell line. Cells were cultured using standard conditions and techniques. Cell viability and GFP-reporter fluorescence were observed using a fluorescent microscope.

**Results:**

We obtained a set of plasmids encoding dCas9-KRAB-MeCP2 repressor under the control of different promoters (hEF1a, hPGK1, mPGK1, hSYN2, synthetic TRE). Non-toxic expression of the CRISPR-repressor was achieved using tetracyclin controllable TRE promoter. Moreover, the Epstein-Barr virus origin of replication (oriP) and its regulator EBNA were introduced to make the self-replicating plasmid. High activity of CRISPR-repressor was confirmed on a schizophrenia-risk gene DDC encoding L-DOPA decarboxylase catalyzing the last step of dopamine biosynthesis.

**Conclusions:**

We have created a plasmid encoding the non-toxic and effective CRISPR repressor encoded by a self-replicating plasmid. The study was supported by the grant from the Russian Science Foundation №21-15-00124, https://rscf.ru/project/21-15-00124/.

**Disclosure:**

No significant relationships.

